# Examining mystical experiences as a predictor of psilocybin-assisted psychotherapy for treatment-resistant depression

**DOI:** 10.1177/02698811251346697

**Published:** 2025-07-01

**Authors:** Ryan M Brudner, Erica Kaczmarek, Marc G Blainey, Christian Schulz-Quach, Shakila Meshkat, Zoe Doyle, Orly Lipsitz, Hilary Offman, Rickinder Sethi, Geneva Weiglein, Roger S McIntyre, Joshua D Rosenblat

**Affiliations:** 1 Mood Disorder Psychopharmacology Unit, UHN, Toronto, ON, Canada; 2 Canadian Rapid Treatment Centre of Excellence, Mississauga, ON, Canada; 3 Department of Pharmacology and Toxicology, University of Toronto, Toronto, ON, Canada; 4 Spiritual Care and Psychotherapy, Martin Luther University College, Waterloo, ON, Canada; 5 Department of Psychiatry, University of Toronto, Toronto, ON, Canada; 6 Department of Psychological Clinical Science, University of Toronto, Toronto, ON, Canada; 7 Brain and Cognition Discovery Foundation, Toronto, ON, Canada

**Keywords:** Major depressive disorder, psilocybin-assisted psychotherapy, mystical experiences, treatment-resistant depression, bipolar disorder

## Abstract

**Background:**

Psilocybin-assisted psychotherapy (PAP) is a promising treatment for various psychiatric disorders. However, the exact biological and psychological mechanisms of action of PAP remain to be determined. Examining predictors of PAP outcomes may help identify necessary processes for positive treatment outcomes. Mystical experiences are considered a key aspect of the subjective effects of ingesting psilocybin. Mystical experiences have been observed to be possibly predictive of positive outcomes in psilocybin treatments. Therefore, some argue that mystical-type experiences are necessary to achieve therapeutic benefits.

**Aims:**

The current study examines mystical experiences as a predictor of antidepressant treatment outcomes in PAP, in a complex clinical sample.

**Methods:**

Participants included 31 individuals with a primary diagnosis of major depressive disorder (MDD) or Bipolar II Disorder (BDII), with treatment resistance to symptoms of their disorder. Participants had one, two, or three PAP treatments with a fixed dose of 25 mg of psilocybin. Depressive symptoms were measured at baseline, at a pre-dose visit and at 2 weeks post-dosing. The presence of mystical experiences was measured on the dosing day after the acute effects had resolved.

**Results:**

For the first psilocybin dose, participants with greater levels of mystical experiences exhibited a greater antidepressant effect from PAP. This effect was not found at the second or third doses.

**Conclusion:**

These results provide preliminary support for the hypothesis that mystical experiences have therapeutic importance in PAP and extend the literature to include a clinical sample of individuals with treatment-resistant depression in the context of MDD or BDII.

## Introduction

Psilocybin has emerged as a promising novel treatment for various domains of psychopathology, especially treatment-resistant major depression (TRD; Pouyan et al., 2022; Rosenblat et al., 2023; Wong et al., 2024). Specifically, psilocybin-assisted psychotherapy (PAP) has been shown to significantly reduce depressive symptoms in those with TRD (Haikazian et al., 2023), with promising preliminary findings also suggesting the efficacy of PAP in the treatment of Bipolar II Disorder (BDII; Aronson et al., 2024), substance-use disorders (van der Meer et al., 2023), eating disorders (Peck et al., 2023) and anxiety disorders (Goldberg et al., 2020), with some evidence of improving trauma-related symptoms (Khan et al., 2022).

PAP involves a dosing session of psilocybin in a controlled setting with a psychotherapist or a psychotherapist dyad present, with preparation and integration psychotherapy sessions surrounding the dosing session. While the exact pharmacodynamic mechanism of action is still unknown, it is understood that psilocybin modulates the serotonergic system as an agonist of the 5HT_2A_ receptor through its active metabolite, psilocin, while also altering key brain circuits, such as the default mode network and the amygdala (Ling et al., 2022). The subjective effects of ingesting psilocybin can include profound experiences with changes in mood, perception, thought and self-experience (Studerus et al., 2011).

As with all treatments, identifying predictors of response and safety is a critical research question. Researchers have started to examine factors that could be moderating psilocybin response and PAP treatment outcomes. These include clinical variables of the participant, such as severity of the disorder (Romeo et al., 2021), patient preparedness for treatment (Modlin et al., 2023), patient characteristics, such as age and personality (Studerus et al., 2012), and the subjective experiences during PAP. Some of these experiences include levels of emotional breakthrough (Dougherty et al., 2023; Roseman et al., 2019), mystical-type experiences (Bogenschutz et al., 2015; Erritzoe et al., 2018; Griffiths et al., 2011, 2016, 2018; Pahnke, 1969; Roseman et al., 2018; Ross et al., 2016, Russ et al., 2019a) and challenging experiences (Barrett et al., 2016; Carbonaro et al., 2016; Haijen et al., 2018; Roseman et al., 2018). Some research using animal models has suggested that the subjective psychedelic experience may not be necessary for psilocybin’s antidepressant effects (Hesselgrave et al., 2021); however, others have argued that these subjective experiences are indeed necessary for psilocybin’s therapeutic benefit in humans (Yaden and Griffiths, 2021).

The presence of a mystical-type experience is associated with positive enduring outcomes such as enhanced personal meaning and spiritual significance (Griffiths et al., 2006), perceived increases in subjective well-being and life satisfaction (Griffiths et al., 2016; Gukasyan et al., 2022; Schmid and Liechti, 2018), positive change in perceived characteristics and attitudes (Russ et al., 2019b), improved depressive and anxiety symptoms (Griffiths et al., 2016; Roseman et al., 2018; Ross et al., 2016), improved addiction behaviour and cravings (Bogenschutz et al., 2015) and even personality changes (Griffiths et al., 2018; MacLean et al., 2011; Weiss et al., 2024a). Moreover, a recent active comparator study comparing PAP to escitalopram found mystical experiences and ego dissolution to mediate the difference in effect observed in PAP versus escitalopram (Weiss et al., 2024b). This study also found mystical experiences, levels of emotional breakthrough, ego dissolution and intense response to music all to moderate the antidepressant effect of PAP. However, the role of mystical experiences in predicting treatment outcomes remains unclear, as some previous research did not find that mystical experiences predicted improvements in depressive symptoms in a wait-list controlled trial (Gukasyan et al., 2022) and in a placebo-controlled trial (Sloshower et al., 2023).

While there remains a great deal of disagreement about how to adequately delineate what a ‘mystical experience’ is and how it can be properly measured (Mosurinjohn, et al., 2023), heretofore most studies of mystical phenomena within PAP have proceeded according to Stace’s (1960) ‘common core’ model of mystical experiences as ‘a self-reported experience of [introvertive or extrovertive types of] unity accompanied by. . . transcendence of time and space, deeply felt positive mood, sacredness, objectivity (noetic quality), ineffability and paradoxicality’ (Barrett and Griffiths, 2018: 396–400). Some researchers have argued for the use of a dichotomous classification for a ‘complete mystical experience’ as meeting this threshold tends to yield distinctive subjective experiences (Barrett et al., 2015; Griffiths et al., 2006; Pahnke, 1969; Russ et al., 2019a). However, this dichotomisation of a continuous outcome has its statistical limitations, reducing variance and thus, power. Although mystical experiences have been shown to moderate positive outcomes in psilocybin treatments, it has yet to be examined prospectively, using the Mystical Experience Questionnaire-30 (MEQ-30) as a predictor of antidepressant response in PAP in a clinical sample of those with TRD. The only study to date that has examined mystical experiences in this sample used a similar construct of oceanic boundlessness, alongside dread of ego dissolution, using the broader Altered States of Consciousness Questionnaire (Dittrich, 1998; Roseman et al., 2018). The subscale of oceanic boundless was constructed similarly to the MEQ-30, drawing influence from Stace’s (1960) ‘mystical experiences’ (Dittrich, 1998). This subscale has also been found to highly correlate with the MEQ-30 (Liechti et al., 2017). Current evidence does not provide clarity as to whether psychedelic experiences are required for psilocybin’s therapeutic effect (Husain et al., 2023; McIntyre, 2023).

A dose of 25 mg of psilocybin is becoming the standard protocol for PAP in the current literature examining PAP as a treatment for major depressive disorder (MDD; Rosenblat et al., 2023). No study to date has reported on the frequency of complete mystical experiences during this standard PAP in a sample of TRD. Whether mystical experiences are beneficial or not, it is pertinent to note what proportion of individuals they occur in during PAP.

In the current exploratory study using data from a previously published wait-list control feasibility trial of PAP (Rosenblat et al., 2024), we examine mystical experiences as a predictor of antidepressant treatment outcomes in PAP in a clinical sample of TRD. It was hypothesised that levels of mystical experience would predict antidepressant outcomes in PAP. Frequencies of dichotomised complete mystical experiences were also examined.

## Methods

### Participants

Participants included 31 individuals with a primary diagnosis of Diagnostic and Statistical Manual of Mental Disorders (Fifth Edition; DSM-5) defined MDD or BDII, with treatment resistance to symptoms of their disorder, as defined as having failed to respond to an adequate dose and duration of at least two guideline-concordant pharmacological treatments for the current major depressive episode. Participants refrained from using augmentation medications (including antidepressants, antipsychotics and ketamine), for the duration of the study. The decision to taper off a current medication was solely up to the participant after consultation with the study doctor (Dr Joshua Rosenblat) and their referring physician. The study doctor could excluded participants for whom clinical assessment indicated that tapering off current medications was not safe or clinically appropriate. Once a participant was successfully tapered off of any current medications, they were eligible to be enrolled in the study. Other exclusion criteria included: being outside of the age range of 18–75, presence of moderate or severe alcohol-use disorder or substance-use disorder in the past 3 months, psychedelic use in the past 12 months, any form of neuromodulation during the trial and any uncontrolled cardiovascular conditions or major concurrent illnesses.

### Procedures

A detailed description of the method of this study can be found in Rosenblat et al. (2024). Written and verbal informed consent was obtained from all participants prior to any study-related procedures. Initial screening consisted of a comprehensive evaluation of physical and mental health by a study doctor, including a clinical diagnostic assessment to confirm eligibility. Participants were randomised to either an immediate treatment group or a waitlist control arm treatment group where they would receive treatment after a 2-week delay.

Synthetic psilocybin was obtained from Usona Institute in powder form, with 25 mg combined with 100 mL of water for each dosing session. Subsection 56(1) exemption and an Import License were obtained from Health Canada to allow for the importation, storage and use of psilocybin for this specific trial. Participants received one, two or three PAP treatments with a fixed dose of 25 mg of psilocybin. Each dose was accompanied by one preparatory therapy session (1–2 h), a supportive dosing session (6–8 h) and two integration therapy sessions (1–2 h each) for a total of approximately 4.5 h of psychotherapy surrounding the dosing session.

All psychotherapy sessions were provided by a multidisciplinary therapist dyad. All therapists underwent an intensive PAP training programme specifically designed for the present trial. The training programme and psychotherapy sessions are described in detail in Rosenblat et al. (2024). Preparatory sessions were focused on psychoeducation, building rapport and setting intentions for treatment. Dosing sessions were provided in a supportive and comfortable environment, which included a place to lie down comfortably, the option of an eye mask, and the option for listening to a preselected music playlist. Food and beverages were provided as needed while avoiding the peak effect period during the first 1–3 h. After the acute effects of psilocybin had resolved in approximately 4–6 h, the therapist dyad and participant had a short debriefing session. The participant was provided with a journal to document any experiences they deemed significant or noteworthy during the psilocybin dosing. The next day, participants returned for an integration session with the therapist dyad to discuss the experience more extensively. A final integration psychotherapy session followed 1 week later, focusing on reflection, meaning-making and revisiting previously set intentions.

In as early as 10 weeks after their first dose, participants were potentially eligible for a second dose based on clinical evidence of all three of the following: clinical benefits from the prior dose (as evidenced by a reduction in *Montgomery-Asberg Depression Rating Scale* (MADRS) score by ⩾50% compared to baseline or a MADRS score < 10), adequate tolerability/safety of prior dose(s), and signs or symptoms of relapse of depression lasting 2 weeks or longer, assessed by the study Doctor. If they met the same criteria after their second dose, they were eligible to receive a third.

All clinical measures were collected at a baseline visit and at 15 subsequent follow-up visits up to 6 months after initial treatment. Primary outcome measures were examined at 2 weeks post-dosing sessions. The questionnaire to assess mystical experiences was completed on dosing days after acute effects had subsided. For detailed results of the primary outcome measures, please see Rosenblat et al. (2024).

### Measures

The MADRS (Montgomery and Asberg, 1979) is a 10-item clinician-rated scale measuring depression severity. Each item is scored from 0 (normal) to 6 (severe) for a total possible score of 60. Higher scores denote greater severity of depressive symptoms. MADRS scores were collected by an independent rater at baseline and at 2 weeks following the first, second and third dosing sessions.

The MEQ-30 (Barrett et al., 2015) is a psychometrically validated 30-item self-report questionnaire evaluating the discrete mystical-type experiences occasioned by psilocybin. Respondents are asked to retrospectively rate their experiences during the session on a Likert scale (0 – None/not at all to 5 – Extreme, more than any other time in my life). The MEQ-30 total score was computed by summing responses to all items. The questionnaire consists of four subscales (Mystical, Positive Mood, Ineffability, and Transcendence of Time and Space). As done in previous studies using the MEQ-30 (Barrett et al., 2015; Griffiths et al., 2006; Russ et al., 2019b), a dichotomous measure of having a ‘complete mystical experience’ is determined by scoring 60% of possible points in each of these subscales. MEQ-30 scores were collected on the day of each psilocybin dosing session after the acute effects subsided, providing a total score after each dose.

### Statistical analysis

At each dose, hierarchical regressions were conducted to determine the incremental effect of MEQ-30 total scores on depression scores at 2 weeks post-dose, above and beyond that explained by pre-dose depression symptom severity. In each model, baseline pre-dose MADRS scores were entered in block 1, and MEQ-30 total scores for the coinciding dosing session (doses 1, 2 or 3) were entered in block 2. Standardised beta coefficients are provided alongside *p*-values. All tests were two-tailed.

Given that some researchers have utilised the construct of a complete mystical experience, frequencies of a complete mystical experience are reported in Table 2. Finally, to assess whether the intensity of mystical experiences significantly differed between PAP doses, a repeated measures analysis of variance (ANOVA) was conducted, with dose number as the independent variable and MEQ-30 total score as the dependent variable. All statistical analyses were conducted using Jamovi and included packages (Fox and Weisberg, 2020; R Core Team, 2021; Singmann, 2018; The Jamovi Project, 2022).

## Results

Participants were recruited and enrolled from 1 November 2021 to 1 February 2023, with all study visits completed by 26 July 2023. Thirty-one participants were enrolled for treatment in the study. One participant withdrew from the study before receiving treatment due to challenges with tapering off antidepressant medications, for a total of 30 participants receiving at least 1 dose of psilocybin. Twenty-eight total participants completed full assessments at 2 weeks post-dose one. Seventeen participants received at least 2 doses and completed full assessments at this time. Five participants received 3 doses and completed the full assessments at this time. Baseline characteristics are summarised in Table 1.

**Table 1.** Baseline participant characteristics.

Baseline characteristic	Overall sample, *N* = 31
Age (in years), mean (SD)	44.4 (13.7)
Female sex	12 (38.7%)
Non-White race/ethnicity	7 (24.1%)
Length of current depressive disorder	
More than 5 years	8 (26%)
Between 2 and 5 years	15 (48%)
Less than 2 years	2 (6.5%)
Undefined	6 (19%)
Primary diagnosis	
Major depressive disorder	27 (87%)
Bipolar II disorder	4 (13%)
Secondary diagnoses	
Anxiety disorder	18 (60%)
ADHD	6 (19.6%)
PTSD	3 (9.6%)
OCD	1 (3.2%)
Gender dysphoria	3 (9.6%)
Eating disorder	3 (9.6%)
Personality disorder	9 (29%)
Previous antidepressant medication trials, mean (SD)	11.27 (5.6)
MADRS at baseline, mean (SD)	30.5 (5.9)

**Figure 1.**
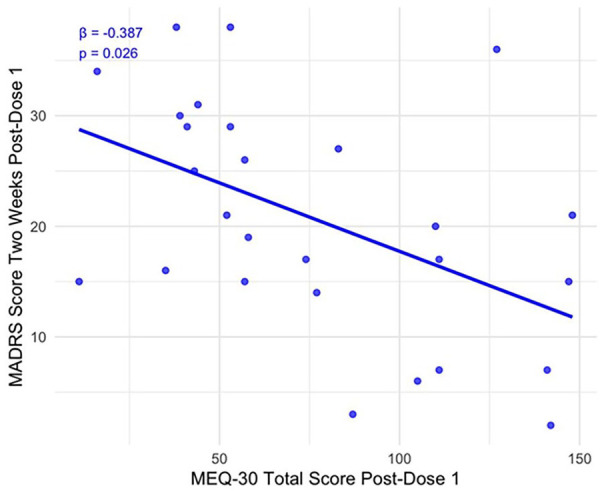
Dose 1 MEQ-30 total scores and MADRS scores 2 weeks post-dose. The *Y*-axis shows actual MADRS scores measured 2 weeks after dose 1. The regression line represents the predictive relationship from the hierarchical regression model, with MEQ-30 total scores and baseline MADRS scores as predictors (β = −0.387, *p* = 0.026).

At dose 1, baseline MADRS scores significantly predicted MADRS scores at week 2, *F*(1, 26) = 8.03, *p* = 0.009. With Dose 1 MEQ-30 total scores entered at block 2, the model was significant, *F*(2, 25) = 7.52, *p* = 0.003. Block 2 explained significantly more variance in week 2 MADRS scores, Δ*R*² = 0.14, *F*(1, 25) = 5.60, *p* = 0.026. In this model, MEQ-30 total scores yielded a standardised beta (β) of −0.387, *p* = 0.026 such that a one SD increase in MEQ-30 scores was associated with a 0.387 decrease in week 2 MADRS scores. This is represented in Figure 1. The models for dose 2, *F*(2, 14) = 1.89, *p* = 0.188, and dose 3, *F*(2, 2) = 1.11, *p* = 0.475, were not significant. Block 2 in each model did not explain more variance in MADRS scores 2 weeks post-dose 2, Δ*R*² = 0.04, *F*(1, 14) = 0.77, *p* = 0.395, or MADRS scores 2 weeks post-dose 3, Δ*R*² = 0.40, *F*(1, 2) = 1.70, *p* = 0.322. A repeated-measures ANOVA showed no difference in MEQ-30 total scores at subsequent doses, *F*(2, 8) = 3.36, *p* = 0.087.

Frequencies of individuals having a complete mystical experience at each dose are summarised in Table 2. When only including those that received at least 2 doses (*n* = 17), 8 (47.1%) participants had a complete mystical experience at dose 2, while only 6 (35.3%) had a complete mystical experience at dose 1. All six of the participants who had a complete mystical experience at dose 1, went on to have a complete mystical experience at dose 2. When only including those that received 3 doses (*n* = 5), 3 (60%) participants had a complete mystical experience at dose 2 and at dose 3, while 1 (20%) participant had a complete mystical experience at dose 1. All three of the participants with a complete mystical experience at dose 2, had a complete mystical experience at dose 3, with one of them having a complete mystical experience at dose 1 as well.

**Table 2.** Mystical experiences at each dose.

Variable	Dose 1, *N* = 28	Dose 2, *N* = 17	Dose 3, *N* = 5
Individuals with a complete mystical experience, count (%)	8 (28.6%)	8 (47.1%)	3 (60%)
MEQ-30 total score, mean (*SD*)	73.1 (42.3)	90.3 (47.6)	77.6 (53.7)
Reduction in pre-dose MADRS, mean (*SD*)	9.5 (9.1)	5.0 (7.9)	6.2 (9.4)

## Discussion

The levels of mystical experiences, represented in total MEQ-30 scores, were not significantly different at subsequent doses. Similarly, the percentage of participants who had a complete mystical experience at dose 1 (28.6%) was descriptively lower than at dose 2 (47.1%) and dose 3 (60%), though these differences were not tested for statistical significance due to the small sample sizes for subsequent doses (see Table 2). This trend was consistent when only including those who had received 2 doses, and, in turn, those who had received 3 doses. Furthermore, when examining the multi-dose cohort of participants, once someone had a complete mystical experience, every participant continued to have further complete mystical experiences at subsequent doses.

Linear regressions revealed that the levels of mystical experience predicted antidepressant effects at dose 1, but not at doses 2 or 3. These findings at dose 1 are in line with previous research demonstrating the importance of mystical experiences in therapeutic outcomes (Bogenschutz et al., 2015; Erritzoe et al., 2018; Griffiths et al., 2006, 2016, 2018; Pahnke, 1969; Roseman et al., 2018; Ross et al., 2016, Russ et al., 2019a; Schmid and Liechti, 2018). This study extends these findings to individuals with TRD and individuals with BDII, as both populations were represented in our clinical sample. The lack of significant effects at doses 2 and 3 may reflect several factors. First, the small sample sizes for dose 2 (*N* = 17) and dose 3 (*N* = 5) likely limit statistical power to detect effects, as fewer participants received subsequent doses due to the eligibility criteria for additional dosing. Second, ceiling effects may have played a role, as participants who experienced significant antidepressant benefits or intense mystical experiences at dose 1 may have had reduced variability in outcomes at subsequent doses, diminishing the ability to detect further associations. Alternatively, the non-significant findings could indicate a true null effect, suggesting that mystical experiences may be the most therapeutically relevant at the initial dose, possibly due to novelty or heightened psychological readiness during the first session. These possibilities highlight the need for larger studies to clarify the role of mystical experiences across multiple doses.

There are a few limitations to note. Firstly, the sample size in this study was chosen to test the safety, tolerability, and effect of PAP in a waitlist-controlled clinical trial. This resulted in a small sample size for the exploratory analyses in the current paper. A larger sample size, especially for those receiving multiple doses of PAP, would provide more adequate power to detect effects of mystical experiences on treatment outcomes. Second, while these findings point to the importance of mystical experiences as captured by the MEQ-30, there are many other constructs subsumed into the acute subjective experience during PAP that may be relevant to treatment outcomes. These include levels of emotional breakthrough, ego dissolution, music impact, visual imagery, positively valenced experiences, priming from a participant’s culture or media consumption and idiosyncratic occurrences in preparation sessions. It is possible that the MEQ-30 elucidates just one of many aspects that make up the acute subjective experience of PAP. Future studies examining PAP should incorporate multiple measures of the acute subjective experience. Third, we did not apply corrections for multiple comparisons when testing the relationship between mystical experiences and antidepressant outcomes across 3 doses. This increases the risk of a Type I error, meaning the significant finding at dose 1 could potentially be an artefact, though its consistency with prior literature supports its plausibility. Fourth, the correlational nature of the current analyses limits causal inferences. A well designed mediation analysis would help elucidate causal effects of mystical experiences on antidepressant outcomes, as recently done in a study examining unique mechanisms in PAP (Weiss et al., 2024a). Lastly, it is possible that there are unique aspects of the PAP treatment protocol used that could be impacting results. These can include the music played, the presence of a therapist dyad, the amount of psychotherapy sessions prior and post-dose, and the use of a couch in the therapy room, among others. To address this limitation, future research should examine the specific therapeutic protocols used in PAP and aspects of each one impacting outcomes. Integrity of blinding procedures has been a challenge in clinical trials examining psychedelics. In previous psychedelic clinical trials, many active controls have been tried and have failed at maintaining integrity of the blind, including diphenhydramine (Bogenschutz et al., 2022), methylphenidate (Griffiths et al., 2006), niacin (Ross et al., 2016) and low-dose psilocybin (Griffiths et al., 2016). Though the waitlist-controlled design of this study is a limitation for causal inferences, this design avoids the ongoing challenge of maintaining integrity of the blind. Despite these limitations, the frequencies of complete mystical experiences observed in this study provide hypothesis-driving data. These data justify future research with more suitable designs to examine differences across subsequent doses and between clinical and healthy control samples in the frequencies and levels of mystical experiences during PAP.

Looking ahead, given mystical experiences within PAP are increasingly opening a new ‘frontier where science and spirituality are meeting’ (Richards, 2015: 211–212), future research in this area must be careful not to bypass the deeply sophisticated nature of mysticism as studied by scholars of religion/theology and by expert practitioners of mystical contemplation within Christianity, Islam, Judaism, Hinduism and Buddhism. As highlighted by Mosurinjohn et al. (2023), current psychedelic research lacks historical and cultural contexts, limiting our current conceptualisations and assessments of this nuanced construct. Considering the accumulating evidence suggesting correlations between mystical experiences and therapeutic benefits, these results highlight how ‘the classic psychedelics sit at the nexus of biological, psychological, social and spiritual perspectives on the fundamental existential problems’ of the human condition, connoting that ‘death, the inevitability of suffering, and the search for meaning are not just related to depression, but probably contribute to a wide variety of other mental health difficulties’ (Rucker, 2019: 74). Indeed, if studies continue to find that mystical experiences predictably lead to improved psychopathological outcomes, it would be wise for scientists to begin consulting and learning from established non-scientific practices. These include indigenous, shamanic and religious traditions of psychedelic ceremonies that comprise well-honed ritual techniques for encouraging, shaping and optimising experiences currently characterised, for lack of a better term, as mystical (Blainey, 2021).

In summary, this article extends the current literature examining how mystical experiences predict antidepressant effects in a clinical sample of individuals with TRD. Levels of mystical experience predicted antidepressant outcomes at the first dose in PAP. However, this relationship was not significant for the second or third doses. These findings highlight the potential importance of mystical experiences in PAP while underscoring the need for larger studies to clarify their role in multiple-dose protocols.
